# Coherent optical interconnects using Fermat number transform and hollow core fibre

**DOI:** 10.1038/s44172-025-00505-3

**Published:** 2025-09-29

**Authors:** Siyu Chen, Zheli Liu, Can Zhao, Mingming Zhang, Peng Li, Lei Zhang, Jie Luo, Zihe Hu, Can Chen, Xuchen Hua, Xianqiao Liao, Zhiyong Zhao, Ming Tang

**Affiliations:** 1 Wuhan National Lab for Optoelectronics (WNLO) & National Engineering Research Centre of Next Generation Internet Access-system (NGIA), School of Optical and Electronic Information, Huazhong University of Science and Technology, Wuhan, China; 2 State Key Laboratory of Optical Fibre and Cable Manufacture Technology, Yangtze Optical Fibre and Cable Joint Stock Limited Company (YOFC), Wuhan, China; 3 Hubei Optical Fundamental Research Centre, Wuhan, China

**Keywords:** Fibre optics and optical communications, Electrical and electronic engineering

## Abstract

With the exponential growth of artificial intelligence-driven data centre traffic, next-generation data centre optical interconnects must deliver high-speed data transmission while ensuring low latency and power consumption. Here, we present an ultra-simple low-latency self-homodyne coherent interconnect solution through anti-resonant hollow core fibre and leverages the Fermat number transform to implement the entire digital signal processing. The Fermat number transform eliminates the round-off errors prevalent in the fast Fourier transform through modulo operations and replaces computationally intensive multiplications with simple cyclic shift and addition operations. As a proof of concept, we demonstrate bidirectional transmission through a 5.1-km anti-resonant hollow core fibre, achieving a data rate of 448 Gb·s^-1^. Our proposed scheme reduces complexity of digital signal processing by 90%, whereas the integration of the anti-resonant hollow core fibre reduces the propagation latency by 28.4%. This work establishes a promising path to push the energy-efficiency boundary of coherent structure and enables large scale deployment of coherent optical interconnects.

## Abstract (web summary)

Siyu Chen, Zheli Liu and colleagues propose a holistic co-design optical communication scheme based on the self-homodyne coherent structure, anti-resonant hollow core fibre, and Fermat number transform. This scheme provides a promising path for the realization of high-speed data centre optical interconnect with low power consumption and low latency.

## Introduction

**Fig. 1:**
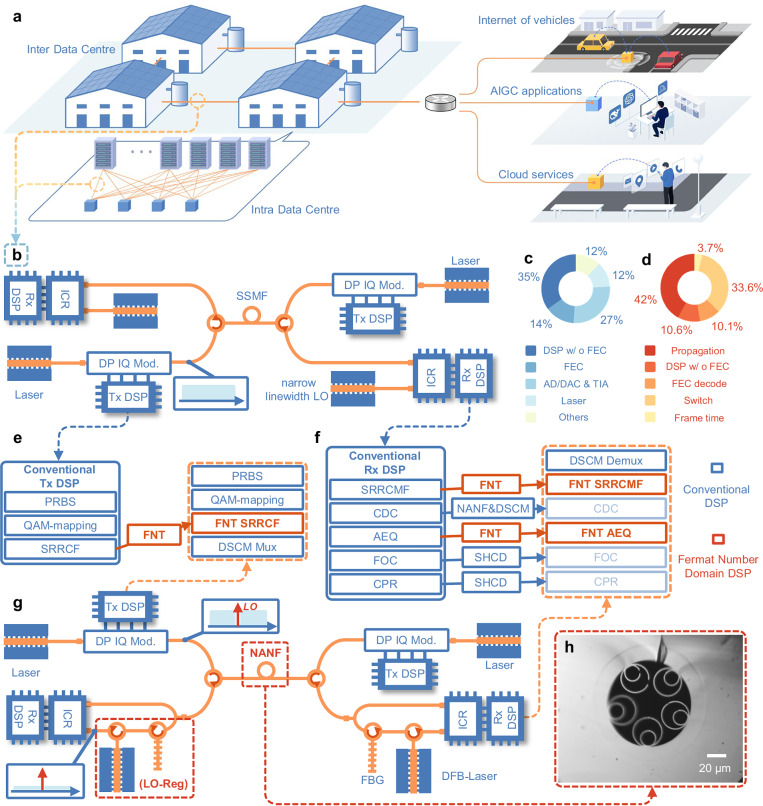
Concept and architecture of the FNT-SHCD scheme. This scheme is designed to address the power consumption and latency challenges faced by conventional coherent technologies in next-generation intra-/inter data centre networks. **a** Intra-/inter data centre optical networks undertake large amounts of data traffic, supporting the internet of vehicles, cloud services, and AIGC training and application processes. **b** Architecture of conventional single-carrier coherent technology in DCNs. **c** Power consumption distribution of the coherent optical module. Others mainly involve power conversion loss. **d** Link latency distribution of the conventional coherent optical module using SSMF. Frame time: the time used to transmit and receive a MAC frame; Switch: Ethernet switches. **e** The proposed FNT-based transmitter (Tx) DSP strategy. **f** The proposed FNT-based receiver (Rx) DSP strategy. The SHCD structure eliminates FOC and CPR algorithms. The use of NANF and DSCM technologies eliminates the CDC block and unlocks the FNT-based AEQ algorithm. By integrating FNT into Nyquist filtering and AEQ blocks, the ultra-simple DSP flow is achieved. **g** The FNT-SHCD architecture for bidirectional transmission: a bias point offset is adopted to insert the LO component into the guard interval of the DSCM signal, and FBG and OIL are used to achieve LO regeneration. **h** The internal microstructure of the NANF with five annular glass tubes. FNT: Fermat number transform; AIGC: artificial intelligence-generated content; DCN: data centre network; DP IQ Mod.: dual polarization in-phase/quadrature modulator; SSMF standard single-mode fibre, LO local oscillator, ICR integrated coherent receiver, DSP digital signal processing, FEC forward error correction, TIA trans-impedance amplifier, ADC analogue-to-digital converter, DAC digital-to-analogue converter, MAC media access control, SHCD self-homodyne coherent detection, FOC frequency offset compensation, CPR carrier phase recovery, NANF nested anti-resonant nodeless fibre, DSCM digital subcarrier multiplexing, CDC chromatic dispersion compensation, AEQ adaptive equalization, PRBS pseudo-random binary sequence, SRRCF square root raised cosine filtering, SRRCMF square root raised cosine matched filtering, DSC Mux/Demux digital signal subcarrier multiplexing/demultiplexing, FBG fibre Bragg grating, OIL optical injection locking, DFB distributed feedback.

Data centres have emerged as essential infrastructures, driven by the explosive growth of Internet of vehicles, cloud services and artificial intelligence-generated content applications based on large language models in recent years^1,2^. These applications have led to a large amount of data traffic on data centre networks (DCNs); intra-data centre traffic typically spanning <2 km accounts for more than 70% of the total internet traffic. To meet the escalating demands for computing power (Fig. 1a)^3,4^, local data centre interconnects (DCIs) are needed to connect to nearby edge data centres within 10 km, forming a super data centre. These developments impose stringent trade-offs and challenges on next-generation DCIs in terms of speed, power consumption, and latency. Emerging high-capacity DC optical interconnects within a speed of 1.6 Tb·s^−1^/3.2·Tb s^−1^ and a distance range of 500 m to 10 km will soon become the mainstream solution to support large-scale data interactions^5^. However, these large-scale, latency-sensitive applications further intensify the requirements for the power consumption and ultra-low link latency of DCIs^6^. Moreover, the fibre and spectrum resources involved in DCs are limited, necessitating spectrally efficient bidirectional (BiDi) transmission at a single wavelength.

Owing to its relatively high receiver sensitivity and spectral efficiency (SE), as well as its ability to compensate for various types of damage, the conventional coherent structure (Fig. 1b) exhibits improved tolerance for fibre propagation impacts^7–9^, making it a promising solution for future DCIs. However, the high latency associated with conventional single-mode fibre (SSMF) and power-hungry complex digital signal processing (DSP) technology remains unacceptable for cost and time-sensitive data centre optical interconnects, hindering the deployment of coherent modules in real short-reach applications. The power consumption and link latency distributions of the coherent optical module are shown in Fig. 1c and d, where the DSP alone consumes half of the power consumption and the propagation time consumes nearly half of the link latency^10–12^. Therefore, innovations in converged transmission architectures, advanced DSP techniques, and fibre hardware are imperative to mitigate the power consumption and latency challenges of traditional coherent technology.

Recently, several BiDi self-homodyne coherent detection (SHCD) transmission schemes have been proposed to reduce the power consumption levels and cost^6,13–16^. By transmitting the LO along with the signal, the requirement for high-cost narrow-linewidth lasers is relaxed. However, these schemes require additional fibres or polarization dimensions to transmit the LO, and the wavelengths of the two BiDi transmission directions need to be staggered, which wastes valuable fibre resources and reduces SE. Moreover, these conventional SSMF-based architectures neglect link latency optimization, leading to suboptimal performance in latency-sensitive applications.

For DSP simplification, SHCD systems eliminate the need for carrier recovery, including frequency offset compensation (FOC) and carrier phase recovery (CPR), leaving chromatic dispersion compensation (CDC), adaptive equalization (AEQ), and Nyquist filtering (NF) as the dominant power consumed by DSP due to their convolutional structures. The conventional simplified DSP approach is mainly based on optimizing the structures of algorithms^17,18^. Although these methods have been proven effective, they have now reached a point where further progress is difficult to achieve. The Fermat number transform (FNT) has attracted considerable attention, owing to its ability to achieve fixed-point convolution without any round-off error via modulus constraint and reduce hardware complexity via circular shift operations^19–21^. Therefore, the FNT provides an efficient path for the implementation of DSP in application-specific integrated circuits (ASICs) and field-programmable gate arrays (FPGAs), greatly reducing the power consumption level. Despite these advantages, the inherent limitations in bit-width and transform length currently hinder the direct deployment of FNT in conventional coherent DSP systems, making innovations in transmission architecture necessary for practical implementation of the FNT-based DSP strategy. The two-dimensional FNT (2D-FNT), which extends the transformation length, is widely used to mitigate the inter-symbol interference (ISI) introduced by CD, especially for single-carrier modulations with speeds above 800 Gb s^−1^ in standard single-mode fibre (SSMF), where an extended transformation length is needed. However, compared with FNT, the 2D-FNT introduces additional hardware complexity overhead and overflow risk, resulting in critical and irreversible computational errors.

In this work, we demonstrate an ultra-simple DSP entirely based on FNT through a simplified and low-latency BiDi transmission coherent structure at a single wavelength, empowered by the adoption of nested anti-resonant nodeless fibre (NANF) and SHCD structure. NANF, as a type of anti-resonant hollow core fibre (AR-HCF), relies on a fundamentally different light guiding mechanism that simultaneously eliminates CD and the refractive index limitations of SSMF^22–24^, and has become a breakthrough solution to simplify DSP and reduce propagation latency^25^. In addition, NANF exhibits negligible nonlinearity and Rayleigh scattering compared with solid-core SSMF^26–28^, enabling high launch power BiDi transmission at the same wavelength in a single fibre. To form a spectrally efficient SHCD structure, we adopt a simple bias offset method called residual carrier modulation (RCM)^29^ to generate a direct-current frequency-domain pilot tone (DC-FPT). The signal is distributed in the form of a digital subcarrier multiplexing (DSCM) signal on both sides of the DC-FPT to provide a sufficient guard interval (GI) to prevent interference between the DC-FPT and the signal. The receiver separates a small amount of the signal and deploys a fibre Bragg grating (FBG) filter to obtain pure DC-FPT components. Therefore, by utilizing a cost-effective distributed feedback (DFB) laser for optical injection locking (OIL), a high-quality LO is regenerated, which is homologous to the signal. By leveraging the aforementioned spectrally efficient SHCD architecture and advanced hollow-core fibre technology, we achieve, a DSP implementation entirely based on the hardware-efficient FNT (Fig. 1e and f). Therefore, we designate the holistic co-design of FNT-DSP, SHCD architecture, and AR-HCF as FNT-SHCD. In addition to the necessary forward error correction (FEC) block, the demonstrated design achieves a 90% reduction in the computational complexity of DSP without compromising transmission performance. Furthermore, experimental validation via bidirectional transmission over a single wavelength demonstrates a 28.4% decrease in the end-to-end latency and a 1.27-dB improvement in the receiver sensitivity. This work establishes a benchmark for next-generation high-speed, low-power, and low-latency optical interconnects in the age of artificial intelligence.

## Results

### FNT-SHCD architecture

The core of the FNT-SHCD structure lies in the slight bias offset of the modulator in the transmitter and the OIL process of the receiver (Fig. 1g). By using DSCM technology, a DC-FPT is inserted between signals located within a certain protection interval. The DC-FPT generation process relies mainly on slightly deviating the bias voltage of the modulator from the null point (*P*_null_). Since the DC-FPT is used for LO regeneration in this work, the carrier-to-signal power ratio (CSPR) is used in this article instead of the pilot-to-signal power ratio (PSR) for characterizing the magnitude relationship between the DC-FPT and the signal power.

Therefore, the DC-FPT and the DSCM signal, which originate from the same laser and propagate along the same optical path, are generated and transmitted to the receiver simultaneously. By deploying OIL, a narrow-linewidth LO can be regenerated. The regenerated LO and signal reach the receiver simultaneously by adjusting the variable optical delay line (VODL). Thus, we achieve SHCD, which eliminates the influences of frequency offsets in the signal and simplifies or even removes the carrier phase recovery algorithm in the DSP. In FNT-SHCD, the signal is located on both sides of the LO in the form of DSCM, which can fully utilize the spectrum resources. Conventionally, spectral separation between the residual optical carrier and signal components induces chromatic-dispersion-driven propagation delay mismatch in higher-order subcarriers, thereby increasing their phase noise. However, the combined effect of the narrow-linewidth characteristic of the regenerated-LO and the low-chromatic-dispersion characteristic of AR-HCF renders the performance degradation negligible (see Supplementary Discussion 1 and Supplementary Fig. 1). Compared with polarization division multiplexing SHCD (PDM-SHCD)^13^ and spatial division multiplexing SHCD (SDM-SHCD)^30^, it has a relatively high SE and does not require additional fibres. Compared with the conventional PDM-SHCD scheme, our proposed FNT-SHCD (with a bandwidth of 60 GHz and a GI of 4 GHz) achieves a higher SE of 86% under the dual polarization 16-quadrature amplitude modulation (16-QAM) format, which is equivalent to that of SDM-SHCD when a 15-core multi-core fibre is used.

### NANF enables low-latency BiDi transmission at a single wavelength

In SSMF, owing to the influence of backscattering, BiDi transmission often operates at different wavelengths. NANF has been proven to have low Rayleigh scattering and nonlinearity^26,27,31^. Therefore, NANF can achieve BiDi transmission at a single wavelength without the requirement of an additional complex wavelength scheduling design.

The internal microstructure of the NANF, which has five annular glass tubes, forms a double-layer nested structure (Fig. 1h). By using a reverse-tapering method and optimizing all splicing parameters, a low splicing loss is achieved between the NANF and SSMF. The sum of the splicing loss and 5.1-km fibre transmission loss is 4.1 dB.

**Fig. 2:**
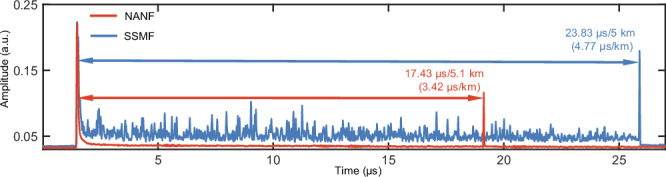
The temporal trace of OTDR in the SSMF and NANF (in one cycle). The propagation latency of NANF is 28.4% lower than that of the SSMF. OTDR optical time-domain reflectometer, SSMF single-mode fibre, NANF nested anti-resonant nodeless fibre.

Owing to the use of NANF, the latency of propagation is greatly reduced^32^. Through optical time-domain reflectometer (OTDR) technology, we obtain the temporal traces of the NANF and SSMF to compare the propagation latency. We compress the time axis to half of the real-time duration to reveal the actual propagation latency. To increase the signal-to-noise ratio (SNR), both traces are averaged 64 times, enabling accurate identification of the peaks corresponding to the front and rear ends of the fibres, as depicted in Fig. 2. The Rayleigh backscattering (RBS) in the NANF is relatively low, so here, we only measure the reflection signal from the end face to determine the propagation latency. By integrating NANF into optical interconnects, a lower RBS signal and a 28.4% lower propagation latency than those of SSMF are achieved.

### FNT-based DSP strategy

**Fig. 3:**
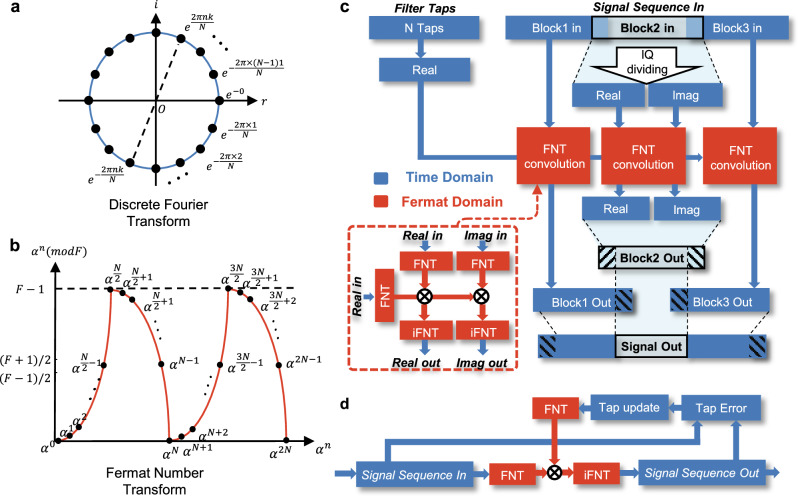
Concept of the FNT-based DSP in the proposed structure. **a** The radix of the FNT is circularly distributed in the finite integer domain, due to the limitation of the modulo operation. Where $F$ represents the modulus value and $\alpha$ represents the radix of the FNT. **b** The radix of the discrete Fourier transform in the domain of complex-valued number. **c** The FNDE architecture is based on the FNT. The real part and imaginary part of the complex-valued sequence are separated and multiplied by the filter taps in the Fermat number domain to implement convolution. **d** The structure of FNT-based adaptive equalization (AEQ). FNT Fermat number transform, DSP digital signal processing, FNDE Fermat number domain equalization.

The number theoretic transform (NTT), as an advanced DSP technique, can achieve convolution results without any round-off error by utilizing the modulo operation. The NTT is defined on the finite integer field, which is realized by the modulo operation on the calculation results. The above transformation has cyclic convolution properties to replace the convolution characteristics of the FFT (Fig. 3a) in the system, if the modulus *F* and radix *α* satisfy the specific expression^33^ (see the “Methods” section for the selection of the modulus *F* and radix *α*). When the radix of the transform *α* = 2^*m*^ and *m* is a positive integer, all complex multiplication operations in the NTT can be replaced with circular shifts that are more efficient for computers or application-specific integrated circuits **(**ASICs). In addition, the modulo operation implemented under Fermat numbers can be achieved through addition. The NTT with the aforementioned characteristics is referred to as the Fermat number transform (FNT), which can be implemented via only addition and cyclic shift operations, eliminating the need for explicit multiplication. Notably, multiplications can be replaced by circular shifts and addition operations when $\sqrt{2}$ is used as the radix of the FNT. During the transformation process, all the data values are confined by using modulo operations; thus, the FNT eliminates the round-off errors that are prevalent in the FFT.

Therefore, a low-complexity FNT with cyclic convolution properties is defined over a finite integer field (Fig. 3b). In this field, cyclic convolutions are implemented efficiently via hardware-friendly cyclic shift operations without any round-off errors.

However, the FNT faces great challenges in the implementation of conventional DSP in the coherent optical communication structure. Owing to the limitations of the finite integer field, overflow occurs once the convolution result exceeds the modulus *F*_*q*_^33^. The mutual limitation between the bit-width and length of the transform must be considered. The relationship between *N*, the signal sequence *x*(*n*) and the tap sequence *h*(*n*) is given as follows^19^:

1 $$|x(n){|}_{\max} {\sum}_{n=0}^{N-1}|h(n)|\le ({F}_{q}-1)/2$$

2 $${{{{\rm{l}}}}{{{\rm{o}}}}{{{\rm{g}}}}}_{2}N+{{{b}}{{w}}}_{h}+{{{b}}{{w}}}_{x}\le {{{{\rm{l}}}}{{{\rm{o}}}}{{{\rm{g}}}}}_{2}({F}_{q}-1)-1$$

where bw_h_ and bw_x_ are the bit-widths of the tap and signal sequences. A low bit-width introduces severe quantization errors. Therefore, it is necessary to determine the required bit-width bw and transform length *N* before integrating the FNT into the algorithms.

The transform length N is also constrained by inequality (1), making it difficult for FNT to support a coherent DSP strategy. With respect to the modulus values *F*_5_ and *F*_6_ (*F*_*n*_ represents ${2}^{{2}^{n}}+1$), the bit-width in the FNT is too large for DSP (*F*_5_ and *F*_6_ require bit-widths of 32 and 64, respectively), whereas *F*_4_ cannot support convolutions with long tap lengths. Therefore, for convolutions with lengths of 128 or longer, the 2D-FNT with modulus *F*_4_ is often used. Our previous work^19,20^ achieved a 256-length convolution via 2D-FNT. Although it provides benefits, the 2D-FNT is still more complex than the FNT. Moreover, owing to the increased convolution length of the 2D-FNT, overflow is more likely to occur, leading to irreversible computational errors.

We implement an ultra-low complexity FNT-based transmitter (Tx) DSP and receiver (Rx) DSP strategies (Fig. 1e and f) through the FNT-SHCD structure. In the conventional DSP strategy, NF (square root raised cosine (SRRC) filtering) is widely used to achieve transmission without ISI, reduce the spectral width of the signal and simultaneously suppress out-of-band noise. Subsequent to the NF block, a static long-tap equalizer is used for the CDC, and then a short-tap AEQ is used for polarization demultiplexing, compensating for the ISI and residual CD. The implementation of the conventional NF, CDC and AEQ blocks relies on time-domain equalization (TDE) based on the finite impulse response (FIR) and frequency-domain equalization (FDE) filters based on the FFT^34^. Although FDE has a lower complexity than TDE for convolutions with long tap lengths, the complexity is still relatively high. Here, we integrate the FNT into both the Tx and Rx DSP strategies by utilizing the structure of Fermat number domain equalization (FNDE) (Fig. 3c). We use an overlap structure to truncate long sequences into short sequences for NF and AEQ.

Notably, the signal is divided into multiple subcarriers in our proposed scheme, thus reducing the baud rate compared with that of the single-carrier scheme. By combining the above method with the use of NANF, the ISI caused by CD is greatly suppressed. Therefore, the AEQ block can deploy the FNT-based AEQ structure^19^ without the CDC (Fig. 3d). Moreover, owing to the use of OIL to perfectly regenerate LO at the receiver, FOC and CPR are eliminated after the LO is aligned with the time delay of the signal path. Therefore, the introduction of the various technologies mentioned above unlocks ultra-simple DSP flow.

### Numerical simulation of the FNT DSP strategy

A numerical simulation is conducted to determine the optimal bit-width for SRRC filtering (the detailed simulation setup is shown in Supplementary Note 1, Supplementary Fig. 2 and Supplementary Table 1). We use the coherent optical communication structure in VPI (VPIphotonics Design Suite v9.9) to transmit 224 (two subcarriers, 2 × 112)-gigabauds (GBaud) DSCM dual-polarization 16-QAM signals for numerical simulation. The fibre length is set to 10 km, the CD coefficient is set to be 4 ps nm^−1^ km^−1^, matched with NANF. We use the concatenated staircase forward error code (C-FEC) with a bit error rate (BER) threshold of 1.21 × 10^−2^ (14.8% overhead^35^), which employs a good trade-off between power consumption and the correction threshold.

**Fig. 4:**
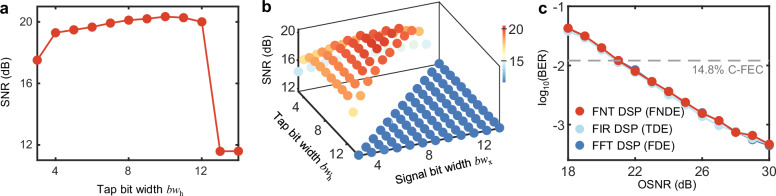
Numerical simulation results. **a** The variation exhibited by the SNR of the system with the bit-widths of the taps in the NF algorithm at the transmitter. **b** The variation exhibited by the SNR of the system with the bit-widths of the signal (bw_*x*_) and taps (bw_*h*_) in the NF block at the receiver. **c** BERs obtained for different OSNRs via multiple DSP schemes in the numerical simulation. Through numerical simulation, it is proven that the performances of the three digital signal processing schemes are identical under 16-bit fixed-point computation. SNR signal-to-noise ratio, NF Nyquist filtering, BER bit error rate, OSNR optical signal-to-noise ratio, C-FEC concatenated staircase forward error code.

For conducting SRRC filtering during Tx DSP, the modulation format of the signal is standard 16-QAM, so the bit-width of the signal is 3. We test the variation exhibited by the SNR with different bit-widths of taps, as shown in Fig. 4a, and the optimal tap bit-width is 10. For the SRRC matched filtering in the Rx DSP, we also test the influences of different tap and signal bit-widths on the SNR. The optimal tap and signal bit-widths are 7 and 8, respectively, as shown in Fig. 4b. The use of a small bit-width will generate quantization noise, leading to a decrease in the SNR. Overflow occurs when a bit-width that is too large is used, resulting in a sharp decrease in the SNR. This phenomenon is consistent with the previous conclusion. We employ multi-subcarrier multiplexing (e.g., 4/8 subcarriers) to achieve higher transmission rates. Crucially, the optimal bit-width distribution identified through the aforementioned point-by-point scanning strategy remains applicable in multi-subcarrier scenarios (see Supplementary Note 2 and Supplementary Fig. 3).

Through numerical simulation, we validate the performance of the proposed FNT-based DSP strategy. All DSP schemes use 16-bit fixed-point computation. Figure 4c indicates that the performances of the FNT, FFT and FIR schemes are almost identical.

### Experimental demonstration of BiDi transmission

**Fig. 5:**
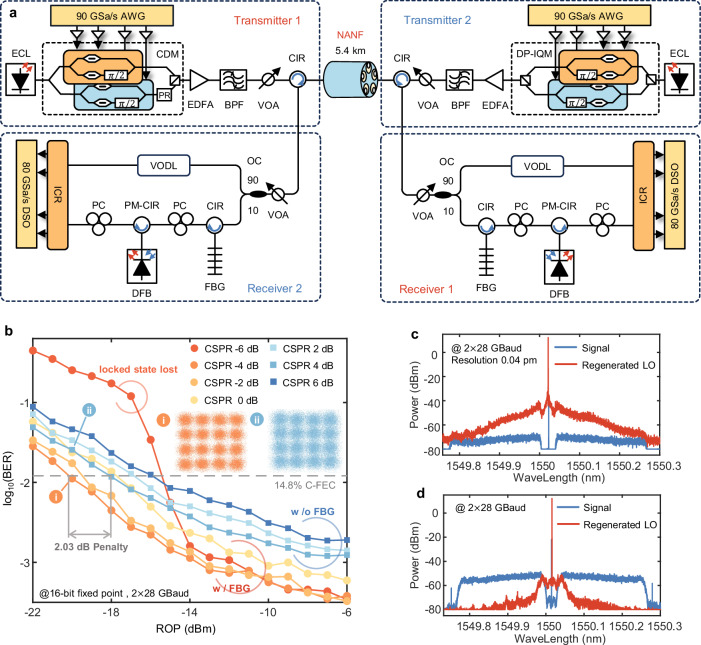
Experimental verification of the proposed scheme. **a** The detailed experimental setup used to verify the performance of the system. **b** BERs obtained for different ROPs under multiple CSPRs. The optimal CSPR of the system with an FBG is smaller than that without the FBG. **c** Spectrum of the OIL input signal and the regenerated LO without the FBG. **d** Spectrum of the OIL input signal and the regenerated LO with the FBG. This finding indicates that the use of the FBG reduces the residual signal component of the regenerated LO. ECL external cavity laser, OC optical coupler, CDM coherent driver modulator, DP-IQM dual-polarization in-phase/quadrature modulator, PR polarization rotator, AWG arbitrary waveform generator, EA electrical amplifier, EDFA erbium doped fibre amplifier, BPF band-pass filter, VOA variable optical attenuator, SSMF standard single-mode fibre, NANF nested anti-resonant nodeless fibre, PC polarization controller, VODL variable optical delay line, PBS polarization beam splitter, PM-CIR polarization-maintaining circulator, ICR integrated coherent receiver, DSO digital storage oscilloscope, BER bit error rate, ROP received optical power, CSPR carrier-to-signal power ratio, FBG fibre Bragg grating, OIL optical injection locking, LO local oscillator.

To validate the proposed scheme, we conduct BiDi transmission over a 5.1-km NANF using the experimental setup depicted in Fig. 5a. As a proof of concept of BiDi transmission at a single wavelength, we set the wavelength at both transmitter 1 and transmitter 2 to 1550.01 nm.

To reduce the residual signal component after conducting OIL, it is necessary to lower the injection power of the OIL to reduce the locking range. However, after the injection power is reduced to approximately −40 dBm, the locked state can be easily lost within minutes. The injection power is controlled simultaneously by the ROP and the CSPR. A high CSPR (>3 dB) results in a large bias offset for the modulator, causing the signal voltage to enter the nonlinear modulation region, introducing device nonlinearity and affecting the quality of the signal. Moreover, a high CSPR indicates elevated LO power levels, which induce a corresponding reduction in the signal power. A low CSPR (less than −6 dB) makes it difficult for OIL to maintain a locked state for a long period under a low ROP. Therefore, we use an FBG to reduce the demand for the CSPR to ensure high-performance transmission.

We obtain the ROP-BER curves at different CSPRs under 56 (two subcarriers, 2 × 28)-GBaud DP-16-QAM, and the results are shown in Fig. 5b. The typical value of CSPR for the proposed scheme is −3 dB. We also test the ROP-BER curve when no FBG filtering is performed in the receiver. According to the results in Fig. 5b, the residual signal component penalizes the receiver sensitivity of 2.03 dB even when we reduce the OIL injection power to enhance the ability to suppress the residual signal. When no FBG filtering is implemented before performing OIL, the increase in the CSPR leads to an increase in the degree of residual signal suppression performed by OIL (the locking range of OIL is shown in the “Methods” section). Therefore, compared with OIL after conducting FBG filtering, the optimal CSPR increases (the optimal CSPR is ~4 dB). Figure 5c and d show the spectra of regenerated LO after OIL with and without an FBG, indicating that the suppression rate of the residual signal increases by more than 16 dB, which can be considered a perfect LO regeneration process.

**Fig. 6:**
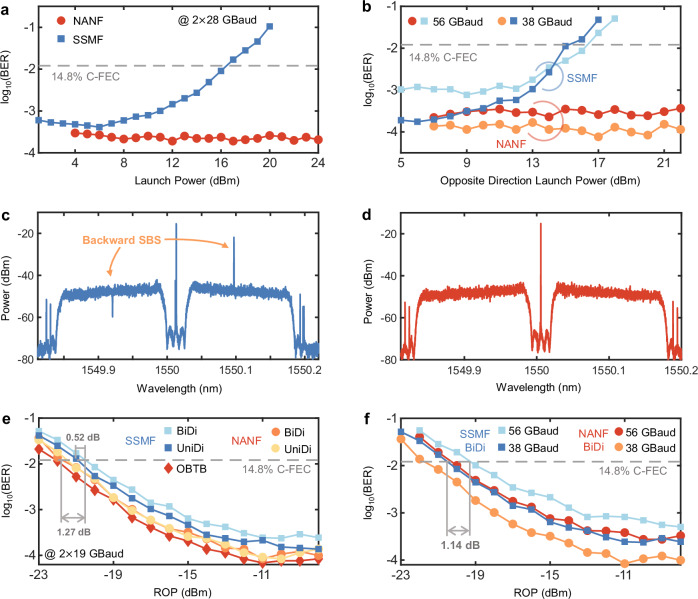
The BiDi transmission performance is achieved through an SSMF and a NANF. **a** BER of the system versus the launch power for different fibres. **b** BER of the system versus the launch power in the opposite direction through different fibres. The penalty imposed on the BiDi transmission performance of SSMF increases when the launch power exceeds 10 dBm in two directions, whereas the transmission performance in the NANF is always consistent. **c** Spectrum of the signal through the NANF in BiDi transmission scenarios. **d** Spectrum of the signal passing through the SSMF in the BiDi transmission scenarios. The performance degradation exhibited by the BiDi transmission scheme used in the SSMF is caused by a power transfer with a frequency interval of 11 GHz, which is attributed to backward simulated Brillouin scattering. **e** BER versus ROP for the NANF and SSMF in the BiDi and UniDi transmissions. **f** BER versus ROP for NANF and SSMF at different baud rates. BiDi bidirectional, SSMF standard single-mode fibre, NANF nested anti-resonant nodeless fibre, BER bit error rate; OBTB optical back-to-back transmission, UniDi unidirectional.

To test the performance of unidirectional (UniDi) and BiDi transmission within the NANF and SSMF in the system, we verify the impacts of the launch power and opposite-direction launch power on the performance of the 5.1-km NANF and 5-km SSMF, obtaining the optimal injection power for different fibres. The low nonlinearity of the NANF can be demonstrated by Fig. 6a. Even with the optimal launch power, a high launch power in the opposite direction still leads to a transmission performance penalty, as shown in Fig. 6b. The spectra produced when the launch power and opposite launch power are set to 6 dBm and 16 dBm are shown in Fig. 6c and d, respectively. In the SSMF, the LO in the opposite direction with high intensity leads to backwards simulated Brillouin scattering, which results in a concavity in the subcarrier spectrum at shorter wavelengths and a protrusion in the subcarrier spectrum at longer wavelengths.

Therefore, when an SSMF is used for BiDi transmission, it is necessary to strictly control the launch power and the opposite launch power. In contrast, the NANF results in much lower fibre nonlinearity, allowing for higher-power BiDi transmission processes, which provide advantages for higher-order modulation formats. Under the optimal launch power (NANF: 22 dBm, SSMF: 6 dBm) and a CSPR of −4 dB, we obtain the optimal ROP-BER curves for the SSMF and NANF in the UniDi and BiDi transmission cases, respectively. In the NANF, the UniDi and BiDi transmission performances are identical, whereas implementing BiDi transmission in the SSMF results in a receiver sensitivity penalty of 0.52 dB at the BER threshold of 1.21 × 10^−3^, as shown in Fig. 6e. The NANF improves the receiver sensitivity of BiDi transmission by 1.27 dB over that attained with the SSMF. A comparison between the performances of the two transmitters is shown in Fig. 6f. The receiver sensitivity of the system is 1.14 dB lower at 56 GBaud than at 38 GBaud.

## Discussion

### The flexibility and scalability of the proposed scheme

The solution we proposed has the ability to achieve various transmission rates and distances. In hollow-core-fibre channels, residual chromatic dispersion (~4 ps/nm/km) becomes the main source of damage. Therefore, for different transmission scenarios, the ISI introduced by chromatic dispersion is considered.

ISI causes an increase in the overlap length of CDC and AEQ, which can be expressed as^36^:

3 $${N}_{{{\mbox{e}}}} > {F}_{{{\mbox{s}}}}T(\,{f}_{\max})=\frac{cLD{f}_{\max}{F}_{{{\mbox{s}}}}}{{{f}_{{{\mbox{c}}}}}^{2}}$$

where *f*_max_ is the maximum frequency of the received signal and *F*_s_ is the sampling rate of the signal. *c*, *L*, *D*, and *f*_c_ are the speed of light, fibre length, CD coefficient and frequency of the optical wave, respectively. *N*_e_ is the number of sampling points affected by inter-symbol interference. Since *F*_s_ is directly proportional to the signal baud rate, the minimum overlap length is proportional to the square of the signal baud rate and the fibre length. As DSCM technology reduces the baud rate of the produced signal, the ISI caused by CD is greatly suppressed. As a verification of the concept, both the signals in the numerical simulation and the experiment conducted in this article are transmitted in the form of two subcarriers.

The tap length of the AEQ is 32, and the overlap length is 16. For 1.6-Tb s^−1^ long-range (LR, 2–10 km) scenarios, deploying two subcarriers in the 16-QAM modulation format can achieve AEQ using FNT. Four subcarriers with rates of 112 GBaud are needed for future 3.2-Tb s^−1^ transmission. For a 1.6-Tb s^−1^ Metro DCI scenario with a typical transmission distance of 80–120 km, the signal can be divided into 8 subcarriers with 28-GBaud rate subcarriers for transmission. Therefore, although our experiment was conducted at a data rate of 400 Gb s^−1^ due to the limitations of the experimental conditions, the experimental results still validate the effectiveness of the simplified coherent structure and DSP strategy we proposed in the data centre optical interconnects with higher transmission capacity in the future.

Our work offers a groundbreaking approach by cross-domain collaboration of transmission architecture, fibre hardware, and algorithms, fundamentally reshaping the foundation of mainstream FFT-based DSP strategy in coherent optical interconnects. Compared with conventional simplification approaches^18,37^, our scheme avoids compromising receiver sensitivity while offering enhanced flexibility. Furthermore, it maintains backward compatibility with conventional simplification approaches, enabling simultaneous deployment with scenario-specific optimizations on the structure of algorithms.

### Hardware complexity and performance estimation of DSP schemes

By calculating the number of real-valued multiplications (RMPs) and real-valued additions (RADs) consumed for each symbol (in one state of polarization), we compare the complexity of the proposed FNT-based NF and AEQ algorithms with those of the FFT, FIR and 2D-FNT schemes.

**Fig. 7:**
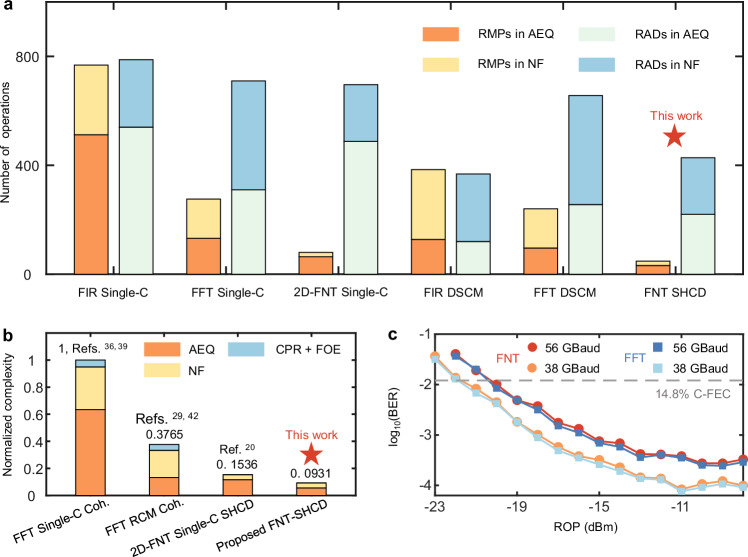
Hardware complexity estimates for different schemes. **a** Statistics of RMPs and RADs in the NF and AEQ blocks based on different techniques. **b** The normalized complexity levels of different schemes. **c** BERs versus ROPs for an NANF implemented via the FNT-DSP scheme and FFT-DSP scheme. RMP real-valued multiplication, RAD real-valued addition, NF Nyquist filtering, AEQ adaptive equalization, CPR carrier phase recovery, FOE frequency offset estimation, Single-C single carrier, DSCM digital subcarrier multiplexing, Single-C Coh. single carrier modulation with coherent structure, RCM Coh. residual carrier modulation with coherent structure, Single-C SDM-SHCD single carrier modulation with the spatial division multiplexing SHCD structure, BER bit error rate, ROP received optical power, FNT Fermat number transform, FFT fast Fourier transform.

The RMPs and RADs of the different schemes are shown in Fig. 7a (refer to the “Methods” section for the workflow of the detailed complexity derivation across different schemes). Compared with the traditional single-carrier FFT scheme, the proposed FNT NF + AEQ scheme reduces the RMPs by 93.75% and the RADs by approximately 45.7%. Compared with the 2D-FNT scheme we previously proposed, the number of RMPs and RADs is further reduced by 40% and 39.5%, respectively. To demonstrate the superiority of the complexity of the proposed scheme, we analyse the overall DSP complexity levels observed under the four schemes. We combine and normalize the complexity of the multipliers and adders by comparing their numbers of half adders, as shown in Fig. 7b. Where a 16-bit Wallace multiplier contains 411 half adders and a 16-bit adder contains 32 half adders^38^. Our solution provides a complexity reduction of ~90% relative to the conventional coherent FFT-based DSP approach^36,39^, excluding the necessary FEC algorithm.

Furthermore, we compare the performance achieved by FNT-DSP and FFT-DSP using data obtained from BiDi transmission in the NANF, as shown in Fig. 7c. Under 16-bit fixed-point computations, the receiver sensitivity of FNT-DSP has almost no penalty relative to that of conventional FFT-DSP.

To further validate the advantages of FNT-DSP, we implement the FNT algorithm on an FPGA platform (XCVU13P-1FLGA2577E). We compare the resource usage between 16-bit FFT and 16-bit FNT for a transform length of 32, as shown in Table 1.

**Table 1:** Resources usage of the FFT and FNT on the FPGA platform

Transformation	CLB LUTs	CLB registers	CARRY8	DSP
FFT (%)	1.39	1.29	1.40	3.42
FNT (include real and imaginary parts) (%)	0.46	0.68	0.88	0.00

*CLB* configurable logic block, *LUT* lookup table, *DSP* digital signal processing.

Notably, the FNT eliminates the use of the valuable DSP resources in the FPGA, and the occupation of lookup table (LUT) and register resources is substantially lower than that of FFT. Although we only validate the complexity of the transform process on the FPGA platform, this comparison of resource usage still provides strong evidence for the superiority of our proposed FNT-DSP scheme in complexity reduction.

Therefore, as a proof of concept, our work validates the feasibility of the holistic co-design combining FNT-DSP, SHCD architecture, and AR-HCF. It concurrently demonstrates that this co-design achieves substantial reductions in transmission latency and power consumption, offering a promising approach for next-generation high-speed coherent optical interconnects.

## Methods

### Generating a direct-current frequency pilot tone

The DC component, originating from the same laser as the signal, is directly induced by the slight bias offset of the modulator. The transfer function of the IQ modulator can be expressed as follows:

4 $${E}_{{{\mbox{out}}}}\left(t\right)= 	 \frac{1}{2}{E}_{{{\mbox{in}}}}[\cos \frac{{{{\rm{\pi }}}}\left({V}_{{{\mbox{sigI}}}}\left({{\mbox{t}}}\right)+{V}_{{{\mbox{offsetI}}}}+{V}_{{{\mbox{biasI}}}}\right)}{2{V}_{{{{\rm{\pi }}}}}} \\ 	 + \cos \frac{{{{\rm{\pi }}}}\left({V}_{{{\mbox{sigQ}}}}\left({{\mbox{t}}}\right)+{V}_{{{\mbox{offsetQ}}}}+{V}_{{{\mbox{biasQ}}}}\right)}{2{V}_{{{{\rm{\pi }}}}}}{{{\mbox{e}}}}^{(\,{{{\mbox{j}}}}{{\varphi }}_{{{\mbox{p}}}})}]$$

where *E*_in_ and *E*_out_ are the input and output optical amplitudes respectively; ${V}_{{{{\rm{\pi }}}}}$ is the half wave voltage of the IQ modulator, *V*_offsetI_ and *V*_offsetQ_ are the offsets of the bias voltage; *V*_biasI_ and *V*_biasQ_ are the bias voltage; *V*_sigI_(*t*) and *V*_sigQ_(*t*) are the signal voltages; ${{\varphi }}_{{{\mbox{p}}}}$ is the phase shift of the quadrature signal. The signal is loaded in the form of digital subcarriers, and Eq. (5) is obtained.

$${V}_{{{\mbox{sig}}}}(t)={V}_{{{\mbox{s}}}}(n{T}_{{{\mbox{s}}}}){{{\mbox{e}}}}^{{{{\rm{j}}}}({\omega }_{i}+{\varphi }_{i}+{\varphi }_{{{\mbox{s}}}}(n{T}_{{{\mbox{s}}}}))}$$

$${V}_{{{\mbox{sigI}}}}(t)={V}_{{{\mbox{s}}}}(n{T}_{{{\mbox{s}}}})\cos ({\omega }_{i}+{\varphi }_{i}+{\varphi }_{{{\mbox{s}}}}(n{T}_{s}))$$

5 $${V}_{{{\mbox{sigI}}}}(t)={V}_{{{\mbox{s}}}}(n{T}_{{{\mbox{s}}}})\sin ({\omega }_{i}+{\varphi }_{i}+{\varphi }_{{{\mbox{s}}}}(n{T}_{{{\mbox{s}}}}))$$

where *V*_s_(*nT*_s_) and ${\varphi }(n{T}_{{{\mbox{s}}}})$ are the modulation amplitude and the phase component of the signal, respectively. ${\omega }_{i}$ is the frequency offset between the *i*th subcarrier and the DC-FPT. If *V*_BiasI_ and *V*_BiasQ_ are set to ${-V}_{\pi }$ and the phase shift ${{\varphi }}_{{{\mbox{p}}}}$ is set to $\frac{\pi }{2}$, then the Taylor expansion can be used to obtain an approximate expression for the output amplitude in the linear modulation region.

6 $${E}_{{{\mbox{out}}}}\left({t}\right)\approx 	 \frac{\pi {{{E}}}_{{{{\mbox{in}}}}}}{4{{{V}}}_{{\pi }}}\left[{{{V}}}_{{s}}\left({{{nT}}}_{{{\mbox{s}}}}\right)\cos ({\omega }_{{{{\mbox{i}}}}}+{\varphi }_{{{{\mbox{i}}}}}+{\varphi }_{{{{\mbox{s}}}}}({{{nT}}}_{{{\mbox{s}}}}))+{{{V}}}_{{{\mbox{offsetI}}}} \right.\\ 	 \left.+{{{\mbox{j}}}}\cdot {{{{\mbox{V}}}}}_{{{\mbox{s}}}}({{{nT}}}_{{{\mbox{s}}}})\sin ({\omega }_{{{{\mbox{i}}}}}+{\varphi }_{{{{\mbox{i}}}}}+{\varphi }_{{{{\mbox{s}}}}}({{{nT}}}_{{{\mbox{s}}}}))+{{{\mbox{j}}}}\cdot {{{{\mbox{V}}}}}_{{{\mbox{offsetQ}}}}\right] \\ 	 =\frac{\pi {{{E}}}_{{{\mbox{in}}}}}{4{{{V}}}_{{\pi }}}\left[{{{V}}}_{s}({{{nT}}}_{{s}}){{{\mbox{e}}}}^{{{{\mbox{j}}}}\left({\omega }_{{{\mbox{i}}}}+{\varphi }_{{{\mbox{i}}}}+{\varphi }_{{{{\mbox{s}}}}}({{{nT}}}_{{s}})\right)}+\sqrt{{{{{V}}}_{{{\mbox{offsetI}}}}}^{2}+{{{{V}}}_{{{\mbox{offsetQ}}}}}^{2}}{{{\mbox{e}}}}^{{{\mbox{j}}}{\varphi }_{{{\mbox{offset}}}}}\right]$$

where ${{\varphi }}_{{{\mbox{offset}}}}$ is the phase of the DC component. The generated DC component is proportional to the square of the bias offsets *V*_offsetI_ and *V*_offsetQ_. This component can be considered DC-FPT. Equation (6) shows that the signal and DC components are separated from each other. Therefore, the LO component is generated between the guard intervals of the DSCM signal with no additional complexity or a receiver sensitivity penalty.

### The detailed parameters of FNT

The transformation and its inverse transformation with lengths of *N* can be defined by the following expressions:

$$X(k)={\sum}^{N-1}_{n=0}x(n)\cdot {\alpha }^{nk}({{{\rm{m}}}}{{{\rm{o}}}}{{{\rm{d}}}}F),k=0,1,\ldots ,N-1$$

7 $$x(n)={\sum}^{N-1}_{n=0}X(k)\cdot {\alpha }^{-nk}({{{\rm{m}}}}{{{\rm{o}}}}{{{\rm{d}}}}F),{n}=0,1,\ldots ,N-1$$

where *x*(*n*) and *X*(*k*) are sequences in the time domain and transformation domain, respectively. *F* and *α* are the modulus and the radix of the FNT, respectively. The modulus *F* is a Fermat number ${F}_{q}={2}^{{2}^{q}}+1$. *F* and *α* must satisfy the following expressions^33^:

8 $$F={{{{p}}}_{{1}}}^{{{{r}}}_{{1}}}{{{{p}}}_{2}}^{{{{r}}}_{2}}\cdots {{{{p}}}_{{{l}}}}^{{{{r}}}_{{{l}}}}$$

9 $${\alpha }^{N}=1({{{\rm{m}}}}{{{\rm{o}}}}{{{\rm{d}}}}{p}_{i}^{{r}_{i}}),i=1,2,\ldots ,l$$

where *p*_*i*_ is the prime factor of *F*. Therefore, we obtain a transformation with convolutional properties in a finite integer domain (Fig. 3b), which is similar to FFT (Fig. 3a) in the complex-valued domain. The radix of the FNT can also be $\sqrt{2}$, which can be expressed as shown below:

10 $${\alpha }_{\sqrt{2}}={2}^{{2}^{q-2}}\cdot ({2}^{{2}^{q-2}}-1)({{{\rm{m}}}}{{{\rm{o}}}}{{{\rm{d}}}}{F}_{q})$$

We denote ${{\alpha }}_{\sqrt{2}}$ as $\sqrt{2}$ because ${{{\alpha }}_{\sqrt{2}}}^{2}=2\left({{\mathrm{mod}}}{F}_{q}\right)$. Thus, $\sqrt{2}$ can also be regarded as the effective radix for eliminating the multiplications of the FNT.

According to the above theory, we provide several typical parameters of the FNT (including the modulus ${F}_{q}$, radix *α*, and transformation length *N* of the FNT) as shown in Table 2.

**Table 2:** Parameters of the FNT

*q*	*F*_*q*_	*N α* = 2	*N α* = $\sqrt{2}$
3	2^8^ + 1	16	32
4	2^16^ + 1	32	64
5	2^32^ + 1	64	128
6	2^64^ + 1	128	256

Here we provide some parameters of the FNT. Note that the moduli *F*_5_ and *F*_6_ introduce high computational bit widths, which are impractical for the DSP hardware implementation. In this paper, we choose *F*_4_ as the modulus of the transformation. *F*_*q*_ is the *q*th Fermat number; *N* is the transform length of the FNT; *α* is the radix of the FNT.

### The detailed complexity calculation in different schemes

The number of complex-valued multiplications (CMPs) (*N*_CMPs-FFT_) and additions (CADs) (*N*_CADs-FFT_) in the FFT are given as

$${N}_{{{\mbox{CMPs}}}-{{\mbox{FFT}}}}=\frac{N}{2}{{{{\rm{l}}}}{{{\rm{o}}}}{{{\rm{g}}}}}_{2}N$$

11 $${N}_{{{{\rm{CADs}}}}-{{{\rm{FFT}}}}}=N{\log }_{2}N$$

where *N* represents the length of the transformation. One CAD is equivalent to two RADs. We employ a simplified scheme utilizing 3 RMPs and 5 RADs for one CMP^34^. Note that the FNT involves only real-valued operations. The numbers of RMPs (*N*_RMPs-FNT_) and RADs (*N*_RADs-FNT_) in the FNT are given as

$${{{\mbox{N}}}}_{{{{\rm{R}}}}{{{\rm{M}}}}{{{\rm{P}}}}{{{\rm{s}}}}-{{{\rm{F}}}}{{{\rm{N}}}}{{{\rm{T}}}}}=0$$

12 $${N}_{{{{\rm{R}}}}{{{\rm{A}}}}{{{\rm{D}}}}{{{\rm{s}}}}-{{{\rm{F}}}}{{{\rm{N}}}}{{{\rm{T}}}}}=N{{{{\rm{l}}}}{{{\rm{o}}}}{{{\rm{g}}}}}_{2}N$$

The algorithmic structure simplification is effective for all the schemes that are based on different transformations. Therefore, we ignore the simplification of the AEQ structure and compare the hardware complexity levels using a 4 × 4 real-valued multiple-input and multiple-output (44RV-MIMO) structure here^40^. 1−*η*_SRRC_ and 1−*η*_AEQ_ are the overlap ratios in the RRC and AEQ blocks, respectively. *ε*_SRRC_ and *ε*_AEQ_ are the corresponding upsampling rates. In Tx DSP, the two subcarriers are upsampled to the sampling rate of an arbitrary waveform generator (AWG), and after performing SRRC filtering based on FNT, subcarrier multiplexing is performed. According to ref. ^41^, for SRRC roll off filtering with a roll off factor (ROF) of 0.05, a 64-tap FDE filter with an overlap length exceeding 20 is generally used to reduce the out-of-band power. Here we use the FNT with a transform length *N* of 64, modulus of *F*_4_ and a radix of $\sqrt{2}$. In Rx, the signal is sampled by a digital sampling oscilloscope (DSO) at the same sampling rate as that of AWG, and performs subcarrier demultiplexing. Subsequently, we can obtain the signal in one subcarrier through FNT-based SRRC matched filtering, while downsampling to 1-sps. Therefore, *ε*_SRRC_, *ε*_AEQ_, *η*_SRRC_, and *η*_AEQ_ are set to 2, 1, 0.5, and 0.5, respectively. The SRRC length *N*_SRRC_ is set to 32, whereas the AEQ length *N*_AEQ_ is set to 16. The transform lengths of SRRC and AEQ algorithms (*M*_FNT-SRRC_, *M*_FFT-SRRC_, *M*_FNT-AEQ_, *M*_FFT-AEQ_) are represented as

$${M}_{{{{\rm{F}}}}{{{\rm{N}}}}{{{\rm{T}}}}-{{{\rm{S}}}}{{{\rm{R}}}}{{{\rm{R}}}}{{{\rm{C}}}}}={M}_{{{{\rm{F}}}}{{{\rm{F}}}}{{{\rm{T}}}}-{{{\rm{S}}}}{{{\rm{R}}}}{{{\rm{R}}}}{{{\rm{C}}}}}=\frac{{N}_{{{\mbox{SRRC}}}}}{{\eta }_{{{\mbox{SRRC}}}}}$$

13 $${M}_{{{{\rm{F}}}}{{{\rm{N}}}}{{{\rm{T}}}}-{{{\rm{A}}}}{{{\rm{E}}}}{{{\rm{Q}}}}}={M}_{{{{\rm{F}}}}{{{\rm{F}}}}{{{\rm{T}}}}-{{{\rm{A}}}}{{{\rm{E}}}}{{{\rm{Q}}}}}=\frac{{N}_{{{\mbox{AEQ}}}}}{{\eta }_{{{\mbox{AEQ}}}}}$$

For the SRRC filtering and matched filtering blocks, taps are constant real-valued number, while the signal is divided into real and imaginary parts as a complex-valued sequence. Consequently, FNT-based SRRC filtering involves eight FNT operations (four at the transmitter and four at the receiver) and multiplication operations between the signal and tap sequences within the Fermat number domain (FND). The numbers of RMPs (*N*_RMPs-SRRC-FNT_) and RADs (*N*_RADs-SRRC-FNT_) in the FNT-based SRRC algorithm are expressed by Eq. (14).

$${N}_{{{\mbox{RMPs}}}-{{\mbox{SRRC}}}-{{\mbox{FNT}}}}=\frac{{\varepsilon }_{{{\mbox{SRRC}}}}\times 4\times {M}_{{{\mbox{FNT}}}-{{{\mbox{SRRC}}}}}}{{N}_{{{\mbox{SRRC}}}}}=\frac{4{\varepsilon }_{{{\mbox{SRRC}}}}}{{\eta }_{{{\mbox{SRRC}}}}}=16$$

$${N}_{{{\mbox{RADs}}}-{{\mbox{SRRC}}}-{{\mbox{FNT}}}}=\frac{{\varepsilon }_{{{\mbox{SRRC}}}}\times \left(8\times {N}_{{{\mbox{RADs}}}-{{{\mbox{FNT}}}}}+8\times {N}_{{{\mbox{RADs}}}-{{{\mbox{FNT}}}}-\sqrt{2}}\right)}{{N}_{{{\mbox{SRRC}}}}}$$

14 $$=8\times \frac{{\varepsilon }_{{{\mbox{SRRC}}}}}{{\eta }_{{{\mbox{SRRC}}}}}\times {{{{\rm{l}}}}{{{\rm{o}}}}{{{\rm{g}}}}}_{2}\left(\frac{{N}_{{{\mbox{SRRC}}}}}{{\eta }_{{{\mbox{SRRC}}}}}\right)+8\times \frac{{\varepsilon }_{{{\mbox{SRRC}}}}}{2{\eta }_{{{\mbox{SRRC}}}}}=208$$

where ${N}_{{{\mbox{RADs}}}-{{\mbox{FNT}}}-\sqrt{2}}$ represents the number of additional RADs when we use ${{\alpha }}_{\sqrt{2}}$ as the radix of FNT.

Similarly, the FFT-based SRRC employs 8 FFT processes. Considering that performing FFT on real-valued sequences reduces computational complexity, the RMPs (*N*_RMPs-SRRC-FFT_) and RADs (*N*_RADs-SRRC-FFT_) in FFT-based SRRC are expressed as

$${N}_{{{\mbox{RMPs}}}-{{\mbox{SRRC}}}-{{\mbox{FFT}}}}=3\times \frac{{\varepsilon }_{{{\mbox{SRRC}}}}\times \left(8\times {N}_{{{\mbox{RMPs}}}-{{{\mbox{FFT}}}}}+4\times {M}_{{{\mbox{FFT}}}-{{{\mbox{SRRC}}}}}\times \frac{1}{2}\right)}{{N}_{{{\mbox{SRRC}}}}}$$

$$=24\times \frac{{\varepsilon }_{{{\mbox{SRRC}}}}}{{4\eta }_{{{\mbox{SRRC}}}}}\times {{{{\rm{l}}}}{{{\rm{o}}}}{{{\rm{g}}}}}_{2}\left(\frac{{N}_{{{\mbox{SRRC}}}}}{{2\eta }_{{{\mbox{SRRC}}}}}\right)+6\times \frac{{\varepsilon }_{{{\mbox{SRRC}}}}}{{\eta }_{{{\mbox{SRRC}}}}}=144$$

$${N}_{{{\mbox{RADs}}}-{{\mbox{SRRC}}}-{{\mbox{FFT}}}}=\frac{2\times {\varepsilon }_{{{\mbox{SRRC}}}}\times 8\times {N}_{{{\mbox{RADs}}}-{{{\mbox{FFT}}}}}}{{N}_{{{\mbox{SRRC}}}}}+\frac{5}{3}\times {N}_{{{\mbox{RMPs}}}-{{\mbox{SRRC}}}-{{{\mbox{FFT}}}}}$$

$$=16\times \frac{{\varepsilon }_{{{\mbox{SRRC}}}}}{{2\eta }_{{{\mbox{SRRC}}}}}\times {\log }_{2}\left(\frac{{{{N}}}_{{{\mbox{SRRC}}}}}{{2\eta }_{{{\mbox{SRRC}}}}}\right)+10\times \frac{{\varepsilon }_{{{\mbox{SRRC}}}}}{{\eta }_{{{\mbox{SRRC}}}}}\times {\log }_{2}\left(\frac{{{{N}}}_{{{\mbox{SRRC}}}}}{{2\eta }_{{{\mbox{SRRC}}}}}\right)+10\times \frac{{\varepsilon }_{{{\mbox{SRRC}}}}}{{\eta }_{{{\mbox{SRRC}}}}}$$

15 $$=18\times \frac{{\varepsilon }_{{{\mbox{SRRC}}}}}{{\eta }_{{{\mbox{SRRC}}}}}\times {{{{\rm{l}}}}{{{\rm{o}}}}{{{\rm{g}}}}}_{2}\left(\frac{{N}_{{{\mbox{SRRC}}}}}{{2\eta }_{{{\mbox{SRRC}}}}}\right)+10\times \frac{{\varepsilon }_{{{\mbox{SRRC}}}}}{{\eta }_{{{\mbox{SRRC}}}}}=400$$

where $5/3\times {N}_{{{\mbox{RMPs}}}-{{\mbox{SRRC}}}-{{\mbox{FFT}}}}$ represents the number of RADs introduced by the RMPs in the FFT process.

The RMPs (*N*_RMPs-SRRC-FIR_) and RADs (*N*_RADs-SRRC-FIR_) in FIR-based SRRC are expressed as

$${N}_{{{{\rm{RMPs}}}}-{{{\rm{SRRC}}}}-{{{\rm{FIR}}}}}=4\times {\varepsilon }_{{{{\rm{SRRC}}}}}\times {N}_{{{{\rm{SRRC}}}}}=256$$

16 $${{{\mbox{N}}}}_{{{{\rm{R}}}}{{{\rm{A}}}}{{{\rm{D}}}}{{{\rm{s}}}}-{{{\rm{S}}}}{{{\rm{R}}}}{{{\rm{R}}}}{{{\rm{C}}}}-{{{\rm{F}}}}{{{\rm{I}}}}{{{\rm{R}}}}}=4\times {\varepsilon }_{{{\mbox{SRRC}}}}\times ({{{\mbox{N}}}}_{{{{\rm{S}}}}{{{\rm{R}}}}{{{\rm{R}}}}{{{\rm{C}}}}}-1)=248$$

In the FNT-based AEQ block, 16 real-valued adaptive filters are split into 32 real-valued filters with a bit-width of 8 for convolution. Therefore, the FNT-based AEQ block involves 44 FNT processes. The numbers of RMPs (*N*_RMPs-AEQ-FNT_) and RADs (*N*_RADs-AEQ-FNT_) in the FNT-based AEQ algorithm are expressed as follows:

$${N}_{{{\mbox{RMPs}}}-{{\mbox{AEQ}}}-{{\mbox{FNT}}}}=\frac{{\varepsilon }_{{{\mbox{AEQ}}}}\times 32\times {M}_{{{\mbox{FNT}}}-{{\mbox{AEQ}}}}}{{N}_{{{\mbox{AEQ}}}}}=\frac{32{\varepsilon }_{{{\mbox{AEQ}}}}}{{\eta }_{{{\mbox{AEQ}}}}}=64$$

$${N}_{{{\mbox{RADs}}}-{{{\mbox{AEQ}}}}-{{{\mbox{FNT}}}}}=\frac{{\varepsilon }_{{{\mbox{AEQ}}}}\times \left(44\times {N}_{{{\mbox{RADs}}}-{{{\mbox{FNT}}}}}\right)}{{N}_{{{\mbox{AEQ}}}}}$$

17 $$=44\times \frac{{\varepsilon }_{{{\mbox{AEQ}}}}}{{\eta }_{{{\mbox{AEQ}}}}}\times {{{{\rm{l}}}}{{{\rm{o}}}}{{{\rm{g}}}}}_{2}\left(\frac{{N}_{{{\mbox{AEQ}}}}}{{\eta }_{{{\mbox{AEQ}}}}}\right)=440$$

The FFT-based AEQ block involves 24 FFT processes. The numbers of RMPs (*N*_RMPs-AEQ-FFT_) and RADs (*N*_RADs-AEQ-FFT_) in the FFT-based AEQ algorithm are expressed as follows:

$${N}_{{{\mbox{RMPs}}}-{{\mbox{AEQ}}}-{{\mbox{FFT}}}}=3\times \frac{{\varepsilon }_{{{\mbox{AEQ}}}}\times \left(24\times {N}_{{{\mbox{RMPs}}}-{{\mbox{FFT}}}}+16\times {M}_{{{{\rm{F}}}}{{{\rm{F}}}}{{{\rm{T}}}}-{{\mbox{AEQ}}}}\times \frac{1}{2}\right)}{{N}_{{{\mbox{AEQ}}}}}$$

$$=72\times \frac{{\varepsilon }_{{{\mbox{AEQ}}}}}{{4\eta }_{{{\mbox{AEQ}}}}}\times {{{{\rm{l}}}}{{{\rm{o}}}}{{{\rm{g}}}}}_{2}\left(\frac{{N}_{{{\mbox{AEQ}}}}}{{2\eta }_{{{\mbox{AEQ}}}}}\right)+24\times \frac{{\varepsilon }_{{{\mbox{AEQ}}}}}{{\eta }_{{{\mbox{AEQ}}}}}=192$$

$${N}_{{{\mbox{RADs}}}-{{\mbox{AEQ}}}-{{\mbox{FFT}}}}=\frac{2\times {\varepsilon }_{{{\mbox{AEQ}}}}\times 24\times {N}_{{{\mbox{RADs}}}-{{\mbox{FFT}}}}}{{N}_{{{\mbox{AEQ}}}}}+\frac{5}{3}\times {N}_{{{\mbox{RMPs}}}-{{\mbox{AEQ}}}-{{\mbox{FFT}}}}$$

$$=48\times \frac{{\varepsilon }_{{{\mbox{AEQ}}}}}{{2\eta }_{{{\mbox{AEQ}}}}}\times {{{{\rm{l}}}}{{{\rm{o}}}}{{{\rm{g}}}}}_{2}\left(\frac{{N}_{{{\mbox{AEQ}}}}}{{2\eta }_{{{\mbox{AEQ}}}}}\right)+30\times \frac{{\varepsilon }_{{{\mbox{AEQ}}}}}{{\eta }_{{{\mbox{AEQ}}}}}\times {{{{\rm{l}}}}{{{\rm{o}}}}{{{\rm{g}}}}}_{2}\left(\frac{{N}_{{{\mbox{AEQ}}}}}{{2\eta }_{{{\mbox{AEQ}}}}}\right)+40\times \frac{{\varepsilon }_{{{\mbox{AEQ}}}}}{{\eta }_{{{\mbox{AEQ}}}}}$$

18 $$=54\times \frac{{\varepsilon }_{{{\mbox{AEQ}}}}}{{\eta }_{{{\mbox{AEQ}}}}}\times {{{{\rm{l}}}}{{{\rm{o}}}}{{{\rm{g}}}}}_{2}\left(\frac{{N}_{{{\mbox{AEQ}}}}}{{2\eta }_{{{\mbox{AEQ}}}}}\right)+40\times \frac{{\varepsilon }_{{{\mbox{AEQ}}}}}{{\eta }_{{{\mbox{AEQ}}}}}=512$$

The numbers of RMPs (*N*_RMPs-AEQ-FIR_) and RADs (*N*_RADs-AEQ-FIR_) in the FIR-based AEQ algorithm are expressed as follows:

$${N}_{{{{\rm{R}}}}{{{\rm{M}}}}{{{\rm{P}}}}{{{\rm{s}}}}-{{{\rm{A}}}}{{{\rm{E}}}}{{{\rm{Q}}}}-{{{\rm{F}}}}{{{\rm{I}}}}{{{\rm{R}}}}}=16\times {\varepsilon }_{{{\mbox{AEQ}}}}\times {N}_{{{\mbox{AEQ}}}}=256$$

19 $${N}_{{{{\rm{R}}}}{{{\rm{A}}}}{{{\rm{D}}}}{{{\rm{s}}}}-{{{\rm{A}}}}{{{\rm{E}}}}{{{\rm{Q}}}}-{{{\rm{F}}}}{{{\rm{I}}}}{{{\rm{R}}}}}=16\times {\varepsilon }_{{{\mbox{AEQ}}}}\times ({N}_{{{\mbox{AEQ}}}}-1)=240$$

Since the output of the AEQ block contains signals of two polarization states, the computational complexity of all aforementioned AEQ implementations should be halved to enable fair comparison. In single-carrier implementations, the AEQ transform lengths (${M}_{{{\mbox{FFT}}}-{{\mbox{AEQ}}}}$ and ${M}_{{{\mbox{FNT}}}-{{\mbox{AEQ}}}}$) are baud rate-dependent as shown in Eq. (3). In this work, the AEQ transform length of the single-carrier scheme is set to be 128.

The carrier phase recovery (CPR) algorithms we use in the conventional coherent structure are the Viterbi-and-Viterbi (V&V)^39^ and residual carrier modulation (RCM) schemes^29,42^. Frequency offset compensation (FOC) is implemented by utilizing FFT to the fourth-power of the signal sequence (FFT-based FOC). The length of the RCM block (*N*_RCM_) and the length of the FOC block (*N*_FOC_) are set to be 256. The parameter *L*, which represents the length of the low-pass filter, is configured as 512 according to ref. ^43^.

Consequently, the numbers of RMPs (*N*_RMPs-CPR-V&V_) and RADs (*N*_RADs-CPR-V&V_) in the V&V-CPR algorithm are expressed as follows:

$${N}_{{{{\rm{RMPs}}}}-{{{\rm{CPR}}}}-{{{\rm{V}}}}{\&} {{{\rm{V}}}}}=\frac{3\times 3\times {{N}}_{{{{{\mbox{V}}}}}{\&}{{{\mbox{V}}}}}+3\times {N}_{{{{{\mbox{V}}}}}{\&}{{{\mbox{V}}}}}+3\times {{{N}}}_{{{{\mbox{V}}}}{\&}{{{\mbox{V}}}}}}{{N}_{{{{\mbox{V}}}}{\&}{{{\mbox{V}}}}}}=15$$

20 $${N}_{{{{\mathrm{RADs}}}}-{{{\mathrm{CPR}}}}-{{{\mathrm{V}}}}\& {{{\mathrm{V}}}}}=\frac{\frac{5}{3}\times {{N}}_{{{\mathrm{RMPs}}}-{{{\mathrm{CPR}}}}-{{{\mathrm{V}}}}\& {{{\mathrm{V}}}}}+2\times {{N}_{{{{\mbox{V}}}}\&{{{\mbox{V}}}}}}}{{N}_{{{{\mbox{V}}}}\&{{{\mbox{V}}}}}}=27$$

The numbers of RMPs (*N*_RMPs-CPR-RCM_) and RADs (*N*_RADs-CPR-RCM_) in the RCM-CPR algorithm are expressed as follows:

$${N}_{{{{\rm{R}}}}{{{\rm{M}}}}{{{\rm{P}}}}{{{\rm{s}}}}-{{{\rm{C}}}}{{{\rm{P}}}}{{{\rm{R}}}}-{{{\rm{R}}}}{{{\rm{C}}}}{{{\rm{M}}}}}=\frac{15\times {N}_{{{\mbox{RCM}}}}}{{N}_{{{\mbox{RCM}}}}}=15$$

21 $${N}_{{{{\rm{R}}}}{{{\rm{A}}}}{{{\rm{D}}}}{{{\rm{s}}}}-{{{\rm{C}}}}{{{\rm{P}}}}{{{\rm{R}}}}-{{{\rm{R}}}}{{{\rm{C}}}}{{{\rm{M}}}}}=\frac{8\times {N}_{{{\mbox{RCM}}}}+L}{{N}_{{{\mbox{RCM}}}}}=10$$

The numbers of RMPs (*N*_RMPs-FFT-based-FOC_) and RADs (*N*_RADs-FFT-based-FOC_) in the FFT-based FOC algorithm are expressed as

$${N}_{{{{\rm{R}}}}{{{\rm{M}}}}{{{\rm{P}}}}{{{\rm{s}}}}-{{{\rm{F}}}}{{{\rm{F}}}}{{{\rm{T}}}}-{{{\rm{b}}}}{{{\rm{a}}}}{{{\rm{s}}}}{{{\rm{e}}}}{{{\rm{d}}}}-{{{\rm{F}}}}{{{\rm{O}}}}{{{\rm{C}}}}} 	= \\ 	\frac{3\times 3\times {N}_{{{\mbox{FOC}}}}+3\times \frac{{N}_{{{\mbox{FOC}}}}}{2}{{{{\rm{l}}}}{{{\rm{o}}}}{{{\rm{g}}}}}_{2}{N}_{{{\mbox{FOC}}}}+3\times {N}_{{{\mbox{FOC}}}}}{{N}_{{{\mbox{FOC}}}}}=24$$

22 $${N}_{{{{\rm{R}}}}{{{\rm{A}}}}{{{\rm{D}}}}{{{\rm{s}}}}-{{{\rm{F}}}}{{{\rm{F}}}}{{{\rm{T}}}}-{{{\rm{b}}}}{{{\rm{a}}}}{{{\rm{s}}}}{{{\rm{e}}}}{{{\rm{d}}}}-{{{\rm{F}}}}{{{\rm{O}}}}{{{\rm{C}}}}}=\frac{5}{3}\times {N}_{{{{\rm{R}}}}{{{\rm{M}}}}{{{\rm{P}}}}{{{\rm{s}}}}-{{{\rm{F}}}}{{{\rm{F}}}}{{{\rm{T}}}}-{{{\rm{b}}}}{{{\rm{a}}}}{{{\rm{s}}}}{{{\rm{e}}}}{{{\rm{d}}}}-{{{\rm{F}}}}{{{\rm{O}}}}{{{\rm{C}}}}}=40$$

The numbers of RMPs (*N*_RMPs-FOC-RCM_) and RADs (*N*_RADs-FOC-RCM_) in the RCM-FOC algorithm are expressed as follows:

$${N}_{{{\mbox{RMPs}}}-{{\mbox{FOC}}}-{{\mbox{RCM}}}}=\frac{2\times {N}_{{{\mbox{RCM}}}}{{{{\rm{l}}}}{{{\rm{o}}}}{{{\rm{g}}}}}_{2}{N}_{{{\mbox{RCM}}}}+4\times {N}_{{{\mbox{RCM}}}}}{{N}_{{{\mbox{RCM}}}}}=20$$

23 $${N}_{{{\mbox{RADs}}}-{{{\mbox{FOC}}}}-{{{\mbox{RCM}}}}}=\frac{3\times {N}_{{{\mbox{RCM}}}}{{{{\rm{l}}}}{{{\rm{o}}}}{{{\rm{g}}}}}_{2}{N}_{{{\mbox{RCM}}}}+2\times {N}_{{{\mbox{RCM}}}}}{{N}_{{{\mbox{RCM}}}}}=26$$

From the analysis presented above, we obtain the overall DSP complexity of different schemes, as shown in Fig. 7a and b.

### The detailed experimental setup

An external cavity laser (ECL, ID Photonics, CoBrite Dx4) is used as transmitters 1 and 2. A signal is generated via the Tx DSP algorithms illustrated in Fig. 1e and f. After generating the baseband signal, an AWG (Keysight M8196A), operating at 90 GSa/s, transforms the signal into four analogue tributaries. They are then sent to the coherent driver modulator (CDM, NeoPhotonics Class 60) for modulation and generating the DC-FPT at transmitter 1. Owing to the limitations of the experimental conditions, we use a dual polarization IQ modulator (DP-IQM, Fujitsu FTM7995HN) at transmitter 2. The 56 GBaud electrical DP-16QAM and 38 GBaud electrical DP-16QAM with symbol lengths of 65,536 are assigned to transmitters 1 and 2, respectively. The DSCM signal modulated with the DC-FPT is amplified by an erbium-doped fibre amplifier (EDFA). A bandpass filter (BPF) with a bandwidth of 100 GHz is used for filtering to eliminate the band noise introduced by the EDFA. The VOA at the transmitter is used to control the launch power of the fibre. Two circulators are deployed at both ends of the fibre to ensure that when transmitting the signals of transmitters 1 and 2 via ports 1 and 2 of the circulators, the signals of transmitters 2 and 1 can be received simultaneously via port 3.

At the receiver, a VOA is used to adjust the received optical power (ROP) of the signal. To reduce the signal power penalty, we use a 90:10 coupler to separate a small amount of the signal to regenerate the LO. A pure DC-FPT can be obtained through filtering via an FBG. The centre wavelength of the utilized FBG is 1550.01 nm, which matches that of the DC-FPT, with a 3 dB bandwidth of ~3.5 GHz. After filtering, the obtained DC-FPT is used as the optical seed source for the OIL, and the polarization of the optical seed source is controlled by a PC. A VODL is used to align the transmission delay between the signal and the regenerated LO, avoiding the additional phase noise caused by the relative time delay (RTD). The DSCM signal and regenerated LO are sent directly to the integrated coherent receiver (ICR, NeoPhotonics Class 40). Following coherent detection, the XI, XQ, YI and YQ components are sampled and received by a DSO (LeCory LabMaster 10-36Zi-A) at a sampling rate of 80 GHz for subsequent offline processing. The optical spectrum in the experiment is acquired through an optical spectrum analyser (OSA, APEX technologies, AP2081A) with a resolution of 0.04 pm. All the experimental results are obtained via the proposed FNT-DSP.

### BER characterization

Each BER value is calculated via a of 2^16^ (65,536)-length pseudo-random binary sequence transmitted on subcarrier −1. We determine the performance of the two subcarriers in the experiment, as shown in Supplementary Fig. 4. The performance of the two subcarriers is consistent. Therefore, in the presentation of the above experimental data, we take the BER value of subcarrier −1 to verify the system performance. Notably, to evaluate the transmission performance of two polarization states (*X* and *Y* polarizations), the BER of each subcarrier is defined as the arithmetic mean of the BER values from both polarization states.

### Locking range of optical injection locking

**Fig. 8:**
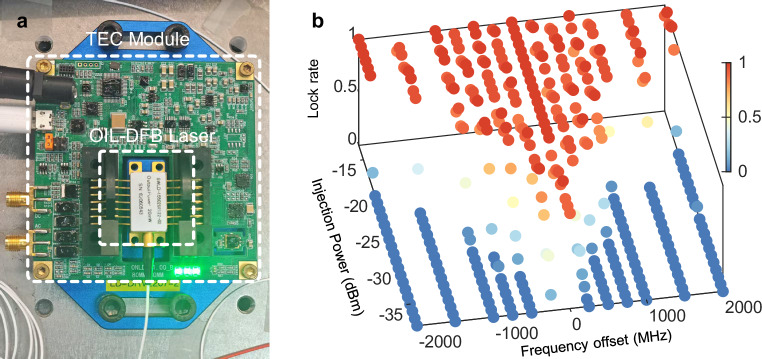
The locking capability of the OIL-DFB laser in the experiment. **a** The picture of the OIL-DFB laser and the corresponding TEC module. **b** Lock rates of OIL for multiple injection powers under different frequency offsets between the LO component and the slave DFB laser. OIL optical injection locking, DFB distributed feedback, TEC thermoelectric cooler.

The regeneration of the LO is the key to the above-described structure. The free-running wavelength of the OIL-DFB laser is adjusted closely to the DC-FPT by utilizing a thermoelectric cooler (TEC) module to realize the regeneration of LO, as shown in Fig. 8a. It is necessary to test the frequency locking range of the OIL-DFB laser used in our experiment. The locking range of the OIL is related to the injection power, satisfying the following expression^44,45^:

24 $$\Delta \omega ={f}_{{{\mbox{d}}}}\sqrt{\frac{{{P}}_{{{\mbox{in}}}}}{{{{P}}}_{{{\mbox{out}}}}}}\cdot \sqrt{1+{\alpha }^{2}}$$

where *f*_d_ is the cavity mode spacing of the OIL-DFB laser; *P*_in_ is the injected power; and *P*_out_ is the output power of the OIL-DFB laser. *α* is the linewidth enhancement factor. $\triangle {\omega }$ represents the drift between the free-running central frequency of the OIL-DFB laser and the frequency of the desired regenerated LO. To distinguish the possibly unstable OIL region, we conduct 20 individual measurements for each set of different frequency offsets and injection powers, and the results are shown in Fig. 8b. With −35 dBm injection power, the tolerant FO range of OIL is −200 to 0 MHz. The asymmetric injection power tolerance at positive and negative frequency offsets is caused by the negative frequency detuning induced by the non-zero linewidth enhancement factor.

## Supplementary information

Supplementary Information

## Data Availability

The data that support the findings of this study are available from the corresponding authors upon reasonable request.

## Code availability

The codes that support the findings of this study are available from the corresponding authors upon reasonable request.
