# Supplementary material for: Coherent optical interconnects using Fermat number transform and hollow core fibre
Source: Commun Eng. 2025 Sep 29;4:169. doi: 10.1038/s44172-025-00505-3 (PMC12480680; doi:10.1038/s44172-025-00505-3)
Supplement: Supplementary file 1 — Supplementary Information [file 44172_2025_505_MOESM1_ESM.pdf]

Supplementary Materials for

"Coherent Optical Interconnects Using Fermat Number Transform and Hollow Core Fibre"

Siyu Chen[1]†, Zheli Liu[1]†, Can Zhao[1], Mingming Zhang[1], Peng Li[2], Lei Zhang[2], Jie Luo[2], Zihe Hu[1], Can Chen[1], Xuchen Hua[1], Xianqiao Liao[1], Zhiyong Zhao[1] and Ming Tang[1,3]*

[1]Wuhan National Lab for Optoelectronics (WNLO) & National Engineering Research Centre of Next Generation Internet Access-system (NGIA), School of Optical and Electronic Information, Huazhong University of Science and Technology, 430074, Wuhan, China.

[2]State Key Laboratory of Optical Fibre and Cable Manufacture Technology, Yangtze Optical Fibre and Cable Joint Stock Limited Company (YOFC), 430073, Wuhan, China.

[3]Hubei Optical Fundamental Research Centre, 430074, Wuhan, China.

*Corresponding author. Email: tangming@mail.hust.edu.cn

†These authors equally contributed to this work.

CONTENTS

Discussion 1 – Phase noise and its influence on self-homodyne coherent detection system 2

Note 1 – Detailed simulation setup deployed to validate the DSP performance 4

Note 2 – Detailed bit-width optimization strategy for multi-subcarrier multiplexing systems. 5

Figures 7

Tables 9

Supplementary References 10

Supplementary Discussion 1: Phase noise and its influence on self-homodyne coherent detection system

In the proposed self-homodyne coherent detection (SHCD) system, we employ multi-subcarrier multiplexing to achieve higher transmission rates. However, higher-order subcarriers exhibit phase noise primarily attributed to the relative time delay (RTD) between the local oscillator (LO) and subcarriers induced by the chromatic dispersion (CD). Therefore, we conduct the quantitative assessment of phase noise impact on spectral-edge subcarriers to validate the robustness of the proposed FNT-SHCD system in high-baud-rate scenarios.

The performance penalty correlates with both laser linewidth and RTD magnitude. Under the constraint of a 0.1-dB receiver sensitivity penalty, the relationship between laser linewidth and mismatch propagation length is shown as (Ref. [S1]):

$$\triangle f \cdot \triangle L \leq 0.01(\mathrm{MHz \cdot m}) \tag{S1}$$

where $\triangle f$ is the linewidth of LO and $\triangle L$ represents the mismatch propagation length between LO and high-order subcarrier. The relationship between the mismatch propagation length and RTD is shown as:

$$\triangle L = \triangle \tau_{\mathrm{RTD}} \cdot \frac{c}{n} \tag{S2}$$

where $\triangle \tau_{\mathrm{RTD}}$ is the relative time delay. c is the speed of light. n represents the refractive index. RTD is induced by chromatic dispersion:

$$\triangle \tau_{\mathrm{RTD}} = DL\triangle\lambda \tag{S3}$$

where D is the chromatic dispersion coefficient, L is the propagation length and $\triangle\lambda$ denotes the wavelength difference between the higher-order subcarrier and the LO. In the experiment, the dispersion coefficient of the AR-HCF was 4 ps/(nm·km) and the refractive index n is 1. For long range (LR) scenario, the propagation distance L is configured to 10 km. Although a DFB laser is employed at the receiver, the linewidth of the regenerated LO is reduced to

approximately 200 kHz through OIL. The optical spectra of the DFB laser before and after OIL are shown in Supplementary Figures 1a and 1b.

Therefore, based on the above theory, the maximum tolerable wavelength difference ($\triangle\lambda$) achievable without employing carrier phase recovery algorithm can be derived:

$$\triangle\lambda \leq \frac{0.01(\mathrm{MHz}\cdot\mathrm{m})\cdot n}{cDL\triangle f} \approx 4.167(\mathrm{nm}) \tag{S4}$$

Given this substantial tolerance for wavelength difference ($\triangle\lambda$), the proposed scheme achieves stable carrier phase recovery capability even for higher-order subcarriers. Consequently, the FNT-SHCD architecture remains feasible in optical interconnects with high baud rate.

Supplementary Note 1: Detailed simulation setup employed to validate DSP performance

The numerical simulation is accomplished in the commercial software VPI Design Suite 9.9. The DSPs are implemented in the software MATLAB R2023b. The detailed setup is shown in Supplementary Figure 2. Due to the current inability of VPI software to simulate the optical injection locking (OIL) processes, we validate the feasibility of the proposed FNT-DSP using a standard coherent optical communication system. The "Set OSNR" module is used to control the optical signal-to-noise ratio (OSNR) of the system. The Supplementary Table 1 presents the detailed simulation parameters. The signal bit-width of receiver side is adjusted by configuring the analogue-to-digital converter (ADC) resolution, while tap bit-widths are controlled within the MATLAB environment.

Supplementary Note 2: Detailed bit-width optimization strategy for multi-subcarrier multiplexing systems.

For higher data rate transmissions, we employ multiple subcarriers (4/8 subcarriers) to achieve the target throughput. Here, we present corresponding simulation results demonstrating the feasibility of the proposed FNT-DSP scheme in the scenarios with higher data rates, as shown in Supplementary Figure 3.

Consistent with our manuscript, the bit-width optimization strategy involves scanning the system performance at different bit-widths for both the transmitter and receiver ends. The objective is to achieve optimal system performance while avoiding overflow in the FNT. Consequently, we performed a point-by-point scanning of the bit-widths for both the signal and the taps in the numerical simulation system for two scenarios: 2 subcarriers (2×112 GBaud) and 4 subcarriers (4×112 GBaud). The results are presented in Supplementary Figures 3a and 3b. These results quantify the impact of quantization error on system performance. The dark blue regions indicate areas where overflow effects are significant. The point-by-point scanning results of system performance versus bit-width indicate that the overflow characteristics of the FNT and optimal bit-width selection remain unaffected by increases in subcarrier quantity or variations in data rate. Consequently, dynamic adjustments to the modulus value F_q and subcarrier configurations are unnecessary in practical deployment scenarios.

Under the optimal bit-width conditions, we simulated the BER versus OSNR performance for two configurations operating at 3.2 $Tb \cdot s^{-1}$: 4 subcarriers (4×112 GBaud) and 8 subcarriers (8×56 GBaud), as shown in Supplementary Figure 3c. The results show that the degradation in receiver sensitivity observed in the 4-subcarrier multiplexing case is attributable to the increase

in system rate. Therefore, the proposed FNT-DSP is capable of supporting both higher data rates and multi-subcarrier multiplexing scenarios.

Supplementary Figures:

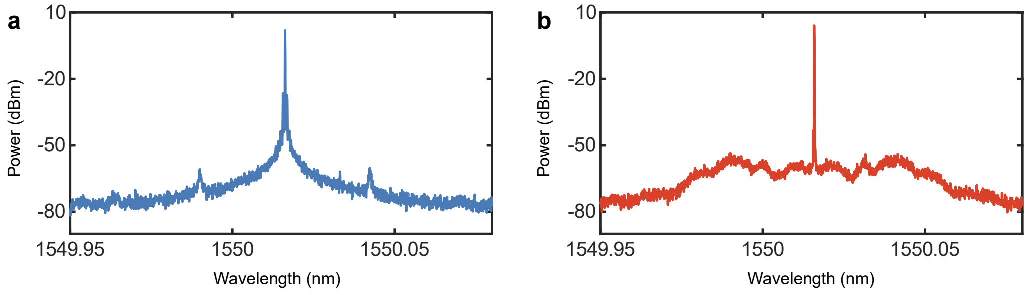

Supplementary Figure 1. Optical spectra of the OIL-DFB laser. a. Optical spectrum of the free-running DFB laser. **b.** Optical spectrum of the DFB laser after OIL. OIL: optical injection locking; DFB: distributed feedback.

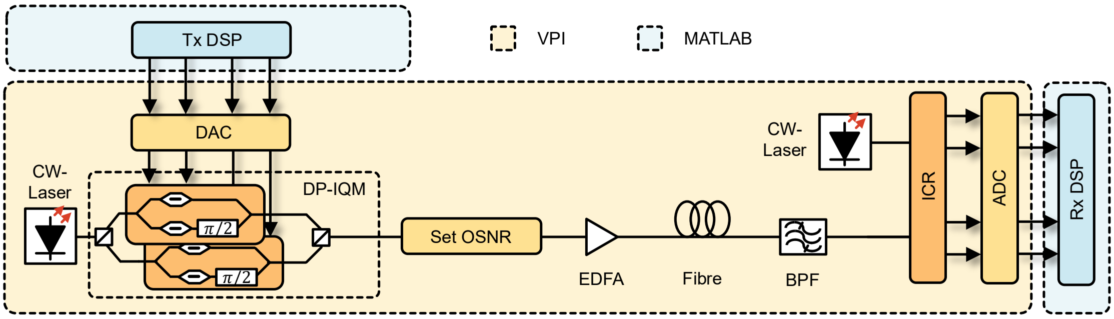

Supplementary Figure 2. The detailed simulation setup. The yellow part is implemented using the software VPI Design Suite 9.9, and the blue part is implemented using the software MATLAB R2023b. Tx: transmitter; Rx: receiver; DSP: digital signal processing; DAC: digital-to-analogue converter; ADC: analogue-to-digital converter; CW: continuous wave; DP-IQM: dual-polarization in-phase/quadrature modulator; OSNR: optical signal-to-noise ratio; EDFA: erbium doped fibre amplifier; BPF: bandpass filter; ICR: integrated coherent receiver.

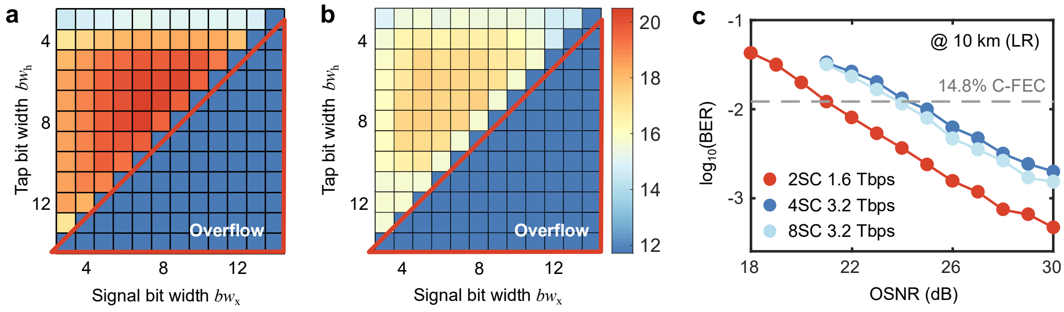

Supplementary Figure 3. The performance of the proposed FNT-DSP for multi-subcarrier multiplexing scenarios. a. Variation of SNR with signal (bw_x) and tap bit-widths (bw_h) in two-subcarrier

multiplexing system (1.6 Tb·s^{-1}). **b.** Variation of SNR with signal and tap bit-widths in four-subcarrier multiplexing system (3.2 Tb·s^{-1}). **c.** BER versus OSNR under multi-subcarrier multiplexing scenarios in the numerical simulation (FNT-DSP). FNT: Fermat number transform; DSP: digital signal processing; SNR: signal-to-noise ratio; OSNR: optical signal-to-noise ratio; SC: subcarrier; LR: long range; C-FEC: concatenated staircase forward error code.

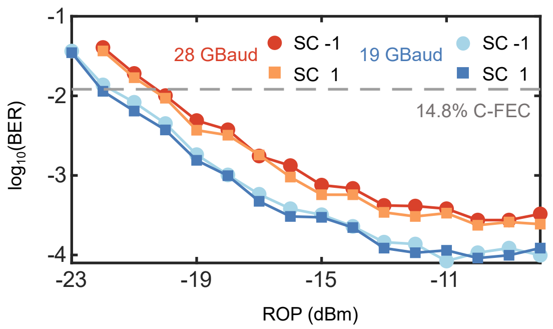

Supplementary Figure 4. BERs versus ROPs for subcarriers -1 and 1. The serial number -1 represents the first subcarrier at a shorter wavelength, and the serial number 1 represents the first subcarrier at a longer wavelength. SC: subcarrier; FEC: forward error correction; BER: bit error rate; ROP: received optical power; C-FEC: concatenated staircase forward error code.

Supplementary Tables:

Supplementary Table 1. The detailed parameters of the simulation system

Laser frequency	193.1 THz
Modulation format	16-QAM
V_π	7 V
Extinction ratio of the modulator	50 dB
Chromatic dispersion of the fibre	4×10^{-6} s·m^{-2}
Length of the fibre	10 km

V_π: half-wave voltage of the modulator.

Supplementary References

[S1] Zhou, X., Gao, Y., Huo, J. & Shieh, W. Theoretical Analysis of Phase Noise Induced by Laser Linewidth and Mismatch Length in Self-Homodyne Coherent Systems. *J. Lightwave Technol.* **39**, 1312–1321 (2021).
